# 2-Meth­oxy-4-(prop-2-en-1-yl)phenyl 2,4-di­chloro­benzoate

**DOI:** 10.1107/S1600536813015675

**Published:** 2013-06-12

**Authors:** Mallikarjuna Rao Pichika, Yew Beng Kang, Seik Weng Ng

**Affiliations:** a Department of Pharmaceutical Chemistry, International Medical University, 126 Jalan Bukit Jalil, 57000 Kuala Lumpur, Malaysia; b Department of Chemistry, University of Malaya, 50603 Kuala Lumpur, Malaysia; c Chemistry Department, Faculty of Science, King Abdulaziz University, PO Box 80203 Jeddah, Saudi Arabia

## Abstract

In the title compound, C_17_H_14_Cl_2_O_3_, the two benzene rings are twisted by 73.6 (2)°. The twist is similar to that found in the unsubstituted compound, *viz.* phenyl benzoate. In the crystal, inversion dimers are linked by pairs of C—H⋯O inter­actions.

## Related literature

For the structure of phenyl benzoate, see: Shibakami & Sekiya (1995 ▶).
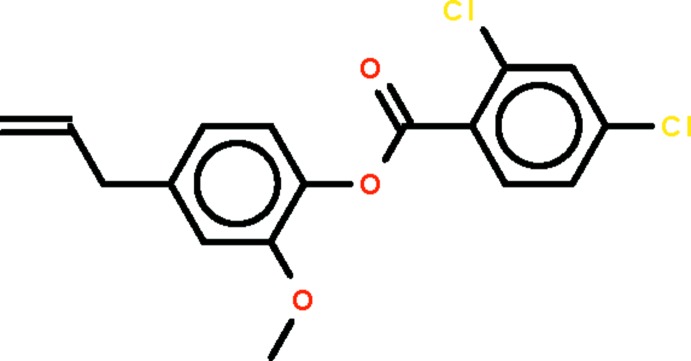


## Experimental

### 

#### Crystal data

C_17_H_14_Cl_2_O_3_
*M* *_r_* = 337.18
Triclinic, 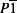

*a* = 7.8805 (8) Å
*b* = 8.4673 (12) Å
*c* = 12.3973 (14) Å
α = 104.166 (11)°
β = 94.502 (9)°
γ = 104.145 (10)°
*V* = 769.29 (16) Å^3^
*Z* = 2
Mo *K*α radiation
μ = 0.43 mm^−1^
*T* = 100 K
0.40 × 0.20 × 0.20 mm

#### Data collection

Agilent SuperNova Dual diffractometer with an Atlas detector
Absorption correction: multi-scan (*CrysAlis PRO*; Agilent, 2013 ▶) *T*_min_ = 0.847, *T*_max_ = 0.919
4945 measured reflections
2703 independent reflections
1818 reflections with *I* > 2σ(*I*)
*R*_int_ = 0.057

#### Refinement

*R*[*F* ^2^ > 2σ(*F* ^2^)] = 0.082
*wR*(*F* ^2^) = 0.269
*S* = 1.09
2703 reflections
199 parameters
H-atom parameters constrained
Δρ_max_ = 1.02 e Å^−3^
Δρ_min_ = −0.64 e Å^−3^

### 

Data collection: *CrysAlis PRO* (Agilent, 2013 ▶); cell refinement: *CrysAlis PRO*; data reduction: *CrysAlis PRO*; program(s) used to solve structure: *SHELXS97* (Sheldrick, 2008 ▶); program(s) used to refine structure: *SHELXL97* (Sheldrick, 2008 ▶); molecular graphics: *X-SEED* (Barbour, 2001 ▶); software used to prepare material for publication: *publCIF* (Westrip, 2010 ▶).

## Supplementary Material

Crystal structure: contains datablock(s) global, I. DOI: 10.1107/S1600536813015675/bt6913sup1.cif

Structure factors: contains datablock(s) I. DOI: 10.1107/S1600536813015675/bt6913Isup2.hkl

Supplementary material file. DOI: 10.1107/S1600536813015675/bt6913Isup3.cml

Additional supplementary materials:  crystallographic information; 3D view; checkCIF report

## Figures and Tables

**Table 1:** Hydrogen-bond geometry (Å, °)

*D*—H⋯*A*	*D*—H	H⋯*A*	*D*⋯*A*	*D*—H⋯*A*
C2—H2⋯O3^i^	0.95	2.52	3.339 (7)	144

Symmetry code: (i) 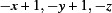.

**Fig. 1.**
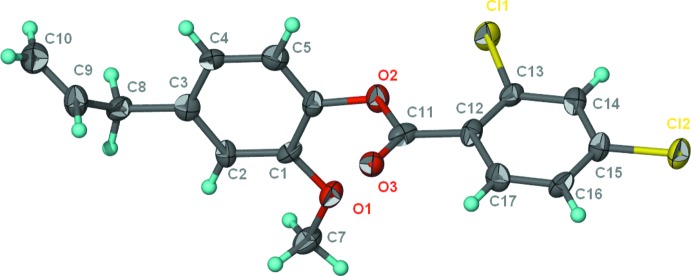
Anisotropic displacement ellipsoid plot (Barbour, 2001) of C17H14Cl2O3 at the 70% probability level; hydrogen atoms are drawn as spheres of arbitrary radius.

## supplementary crystallographic information

### Comment

The title phenyl benzoate (Scheme I, Fig. 1), which possesses an allyl and a methoxy substituent, was synthesized for an evaluation of its pharmaceutical properties as it is an ester derivative of eugenol. The two benzene rings are approximately perpendicular [dihedral angle 73.6 (25) °]. The twist is similar to that found in the unsubstituted compound, phenyl benzoate (Shibakami & Sekiya, 1995).

### Experimental

4-Allyl-2-methoxyphenol (1 mmol), 2,4-dichlorobenzoic acid (1 mmol), diethylazodicarboxylate (2 mmol) and triphenylphosphine (2 mmol) were heated in THF (10 ml) for 2 h. The solid material extracted with dichloromethane. The dichloromethane solution was eluted through a silica gel column by using an *n*-hexane–ethyl acetate (95: 5 *v*/*v*) solvent system. Slow evaporation of the solution yielded large colorless crystals.

### Refinement

H-atoms were placed in calculated positions [C–H 0.95 to 0.98 Å, *U*_iso_(H) 1.2 to 1.5*U*_eq_(C)] and were included in the refinement in the riding model approximation.

The final difference Fourier map had a peak at 1 Å from Cl1.

### Crystal data

**Table:**

C_17_H_14_Cl_2_O_3_	*Z* = 2
*M**_r_* = 337.18	*F*(000) = 348
Triclinic, *P*1	*D*_x_ = 1.456 Mg m^−^^3^
Hall symbol: -P 1	Mo *K*α radiation, λ = 0.71073 Å
*a* = 7.8805 (8) Å	Cell parameters from 1164 reflections
*b* = 8.4673 (12) Å	θ = 3.0–25.0°
*c* = 12.3973 (14) Å	µ = 0.43 mm^−^^1^
α = 104.166 (11)°	*T* = 100 K
β = 94.502 (9)°	Prism, colourless
γ = 104.145 (10)°	0.40 × 0.20 × 0.20 mm
*V* = 769.29 (16) Å^3^	

### Data collection

**Table:**

Agilent SuperNova Dual diffractometer with an Atlas detector	2703 independent reflections
Radiation source: SuperNova (Mo) X-ray Source	1818 reflections with *I* > 2σ(*I*)
Mirror monochromator	*R*_int_ = 0.057
Detector resolution: 10.4041 pixels mm^-1^	θ_max_ = 25.0°, θ_min_ = 3.0°
ω scan	*h* = −9→9
Absorption correction: multi-scan (*CrysAlis PRO*; Agilent, 2013)	*k* = −9→10
*T*_min_ = 0.847, *T*_max_ = 0.919	*l* = −14→9
4945 measured reflections	

### Refinement

**Table:**

Refinement on *F*^2^	Primary atom site location: structure-invariant direct methods
Least-squares matrix: full	Secondary atom site location: difference Fourier map
*R*[*F*^2^ > 2σ(*F*^2^)] = 0.082	Hydrogen site location: inferred from neighbouring sites
*wR*(*F*^2^) = 0.269	H-atom parameters constrained
*S* = 1.09	*w* = 1/[σ^2^(*F*_o_^2^) + (0.1481*P*)^2^ + 0.2039*P*] where *P* = (*F*_o_^2^ + 2*F*_c_^2^)/3
2703 reflections	(Δ/σ)_max_ = 0.001
199 parameters	Δρ_max_ = 1.02 e Å^−^^3^
0 restraints	Δρ_min_ = −0.64 e Å^−^^3^

### Fractional atomic coordinates and isotropic or equivalent isotropic displacement parameters (Å^2^)

**Table:**

	*x*	*y*	*z*	*U*_iso_*/*U*_eq_	
Cl1	0.61313 (17)	0.1630 (2)	0.41224 (12)	0.0305 (5)	
Cl2	1.30151 (17)	0.3673 (2)	0.56261 (11)	0.0286 (5)	
O1	0.5171 (5)	0.5487 (5)	0.1955 (3)	0.0248 (10)	
O2	0.5571 (5)	0.2458 (5)	0.2059 (3)	0.0247 (10)	
O3	0.7704 (5)	0.3027 (5)	0.0993 (3)	0.0244 (10)	
C1	0.4077 (7)	0.4099 (7)	0.1176 (4)	0.0196 (12)	
C2	0.2748 (7)	0.4127 (7)	0.0377 (4)	0.0210 (13)	
H2	0.2580	0.5184	0.0333	0.025*	
C3	0.1662 (7)	0.2644 (8)	−0.0358 (4)	0.0225 (13)	
C4	0.1948 (7)	0.1095 (7)	−0.0313 (5)	0.0232 (13)	
H4	0.1231	0.0072	−0.0820	0.028*	
C5	0.3276 (7)	0.1049 (8)	0.0470 (5)	0.0252 (14)	
H5	0.3464	−0.0005	0.0504	0.030*	
C6	0.4327 (7)	0.2539 (7)	0.1202 (4)	0.0205 (13)	
C7	0.4783 (8)	0.7069 (7)	0.1996 (5)	0.0297 (15)	
H7A	0.5628	0.7976	0.2581	0.045*	
H7B	0.3579	0.7001	0.2170	0.045*	
H7C	0.4876	0.7311	0.1266	0.045*	
C8	0.0147 (7)	0.2725 (8)	−0.1161 (4)	0.0258 (14)	
H8A	−0.0981	0.2270	−0.0902	0.031*	
H8B	0.0251	0.3927	−0.1123	0.031*	
C9	0.0062 (8)	0.1778 (8)	−0.2361 (5)	0.0286 (15)	
H9	0.1073	0.2072	−0.2716	0.034*	
C10	−0.1295 (9)	0.0577 (8)	−0.2954 (5)	0.0335 (15)	
H10A	−0.2329	0.0248	−0.2627	0.040*	
H10B	−0.1250	0.0032	−0.3714	0.040*	
C11	0.7283 (7)	0.2824 (7)	0.1879 (5)	0.0218 (13)	
C12	0.8567 (7)	0.2914 (7)	0.2851 (4)	0.0218 (13)	
C13	0.8232 (7)	0.2503 (7)	0.3850 (5)	0.0209 (13)	
C14	0.9597 (7)	0.2728 (7)	0.4692 (4)	0.0216 (13)	
H14	0.9352	0.2423	0.5363	0.026*	
C15	1.1335 (7)	0.3406 (8)	0.4549 (4)	0.0222 (13)	
C16	1.1726 (7)	0.3802 (7)	0.3571 (4)	0.0237 (13)	
H16	1.2917	0.4228	0.3469	0.028*	
C17	1.0337 (7)	0.3565 (8)	0.2737 (4)	0.0245 (13)	
H17	1.0595	0.3855	0.2063	0.029*	

### Atomic displacement parameters (Å^2^)

**Table:**

	*U*^11^	*U*^22^	*U*^33^	*U*^12^	*U*^13^	*U*^23^
Cl1	0.0151 (8)	0.0452 (11)	0.0321 (9)	0.0026 (7)	0.0048 (6)	0.0172 (7)
Cl2	0.0183 (8)	0.0381 (10)	0.0272 (9)	0.0058 (7)	−0.0021 (6)	0.0082 (7)
O1	0.018 (2)	0.026 (2)	0.027 (2)	0.0043 (18)	−0.0019 (16)	0.0035 (18)
O2	0.019 (2)	0.034 (2)	0.023 (2)	0.0072 (18)	0.0033 (16)	0.0102 (18)
O3	0.021 (2)	0.028 (2)	0.023 (2)	0.0034 (18)	0.0032 (16)	0.0096 (18)
C1	0.016 (3)	0.017 (3)	0.020 (3)	0.000 (2)	0.003 (2)	−0.001 (2)
C2	0.019 (3)	0.029 (3)	0.017 (3)	0.008 (3)	0.003 (2)	0.006 (2)
C3	0.019 (3)	0.030 (3)	0.020 (3)	0.008 (3)	0.009 (2)	0.007 (3)
C4	0.020 (3)	0.021 (3)	0.026 (3)	0.000 (3)	0.003 (2)	0.009 (3)
C5	0.020 (3)	0.027 (3)	0.032 (3)	0.008 (3)	0.007 (2)	0.010 (3)
C6	0.016 (3)	0.026 (3)	0.019 (3)	0.006 (2)	0.002 (2)	0.006 (2)
C7	0.024 (3)	0.021 (3)	0.038 (4)	0.005 (3)	−0.001 (3)	0.000 (3)
C8	0.018 (3)	0.023 (3)	0.033 (3)	0.003 (3)	0.000 (2)	0.007 (3)
C9	0.021 (3)	0.043 (4)	0.025 (3)	0.012 (3)	0.004 (2)	0.012 (3)
C10	0.038 (4)	0.025 (4)	0.034 (4)	0.011 (3)	−0.004 (3)	0.002 (3)
C11	0.017 (3)	0.016 (3)	0.030 (3)	0.005 (2)	0.007 (2)	0.002 (3)
C12	0.018 (3)	0.022 (3)	0.023 (3)	0.008 (2)	−0.001 (2)	0.001 (2)
C13	0.012 (3)	0.021 (3)	0.029 (3)	0.004 (2)	0.002 (2)	0.006 (2)
C14	0.021 (3)	0.024 (3)	0.020 (3)	0.010 (3)	0.001 (2)	0.002 (2)
C15	0.019 (3)	0.030 (3)	0.016 (3)	0.008 (3)	0.001 (2)	0.002 (2)
C16	0.014 (3)	0.027 (3)	0.025 (3)	0.000 (2)	0.002 (2)	0.003 (3)
C17	0.027 (3)	0.031 (4)	0.016 (3)	0.008 (3)	0.005 (2)	0.006 (3)

### Geometric parameters (Å, º)

**Table:**

Cl1—C13	1.736 (5)	C7—H7C	0.9800
Cl2—C15	1.736 (5)	C8—C9	1.494 (8)
O1—C1	1.369 (6)	C8—H8A	0.9900
O1—C7	1.436 (7)	C8—H8B	0.9900
O2—C11	1.357 (6)	C9—C10	1.303 (9)
O2—C6	1.414 (6)	C9—H9	0.9500
O3—C11	1.210 (7)	C10—H10A	0.9500
C1—C6	1.390 (8)	C10—H10B	0.9500
C1—C2	1.393 (7)	C11—C12	1.486 (8)
C2—C3	1.390 (8)	C12—C13	1.393 (8)
C2—H2	0.9500	C12—C17	1.401 (8)
C3—C4	1.397 (8)	C13—C14	1.382 (7)
C3—C8	1.520 (7)	C14—C15	1.393 (8)
C4—C5	1.385 (8)	C14—H14	0.9500
C4—H4	0.9500	C15—C16	1.373 (8)
C5—C6	1.383 (7)	C16—C17	1.386 (8)
C5—H5	0.9500	C16—H16	0.9500
C7—H7A	0.9800	C17—H17	0.9500
C7—H7B	0.9800		
			
C1—O1—C7	115.8 (4)	C3—C8—H8B	108.5
C11—O2—C6	115.7 (4)	H8A—C8—H8B	107.5
O1—C1—C6	116.3 (5)	C10—C9—C8	124.8 (6)
O1—C1—C2	125.6 (5)	C10—C9—H9	117.6
C6—C1—C2	118.1 (5)	C8—C9—H9	117.6
C3—C2—C1	121.5 (5)	C9—C10—H10A	120.0
C3—C2—H2	119.2	C9—C10—H10B	120.0
C1—C2—H2	119.2	H10A—C10—H10B	120.0
C2—C3—C4	119.1 (5)	O3—C11—O2	122.4 (5)
C2—C3—C8	119.7 (5)	O3—C11—C12	123.7 (5)
C4—C3—C8	121.2 (5)	O2—C11—C12	114.0 (5)
C5—C4—C3	120.0 (5)	C13—C12—C17	117.4 (5)
C5—C4—H4	120.0	C13—C12—C11	128.7 (5)
C3—C4—H4	120.0	C17—C12—C11	113.8 (5)
C6—C5—C4	119.9 (5)	C14—C13—C12	121.1 (5)
C6—C5—H5	120.0	C14—C13—Cl1	115.1 (4)
C4—C5—H5	120.0	C12—C13—Cl1	123.8 (4)
C5—C6—C1	121.4 (5)	C13—C14—C15	119.5 (5)
C5—C6—O2	119.1 (5)	C13—C14—H14	120.3
C1—C6—O2	119.3 (5)	C15—C14—H14	120.3
O1—C7—H7A	109.5	C16—C15—C14	121.3 (5)
O1—C7—H7B	109.5	C16—C15—Cl2	120.5 (4)
H7A—C7—H7B	109.5	C14—C15—Cl2	118.2 (4)
O1—C7—H7C	109.5	C15—C16—C17	118.2 (5)
H7A—C7—H7C	109.5	C15—C16—H16	120.9
H7B—C7—H7C	109.5	C17—C16—H16	120.9
C9—C8—C3	114.9 (4)	C16—C17—C12	122.4 (5)
C9—C8—H8A	108.5	C16—C17—H17	118.8
C3—C8—H8A	108.5	C12—C17—H17	118.8
C9—C8—H8B	108.5		
			
C7—O1—C1—C6	−173.9 (5)	C6—O2—C11—O3	−7.7 (7)
C7—O1—C1—C2	5.8 (7)	C6—O2—C11—C12	173.5 (4)
O1—C1—C2—C3	−177.9 (5)	O3—C11—C12—C13	−171.3 (6)
C6—C1—C2—C3	1.7 (8)	O2—C11—C12—C13	7.5 (8)
C1—C2—C3—C4	−1.9 (8)	O3—C11—C12—C17	11.4 (8)
C1—C2—C3—C8	175.7 (5)	O2—C11—C12—C17	−169.9 (4)
C2—C3—C4—C5	1.2 (8)	C17—C12—C13—C14	−0.1 (8)
C8—C3—C4—C5	−176.3 (5)	C11—C12—C13—C14	−177.4 (5)
C3—C4—C5—C6	−0.5 (8)	C17—C12—C13—Cl1	−178.2 (4)
C4—C5—C6—C1	0.4 (8)	C11—C12—C13—Cl1	4.6 (9)
C4—C5—C6—O2	174.5 (4)	C12—C13—C14—C15	1.2 (8)
O1—C1—C6—C5	178.7 (5)	Cl1—C13—C14—C15	179.4 (4)
C2—C1—C6—C5	−1.0 (8)	C13—C14—C15—C16	−2.3 (9)
O1—C1—C6—O2	4.6 (7)	C13—C14—C15—Cl2	179.9 (4)
C2—C1—C6—O2	−175.1 (4)	C14—C15—C16—C17	2.2 (9)
C11—O2—C6—C5	103.7 (6)	Cl2—C15—C16—C17	180.0 (4)
C11—O2—C6—C1	−82.0 (6)	C15—C16—C17—C12	−1.1 (9)
C2—C3—C8—C9	130.2 (6)	C13—C12—C17—C16	0.1 (8)
C4—C3—C8—C9	−52.2 (7)	C11—C12—C17—C16	177.8 (5)
C3—C8—C9—C10	122.7 (7)		

### Hydrogen-bond geometry (Å, º)

**Table:**

*D*—H···*A*	*D*—H	H···*A*	*D*···*A*	*D*—H···*A*
C2—H2···O3^i^	0.95	2.52	3.339 (7)	144
C17—H17···O3	0.95	2.39	2.746 (6)	101

Symmetry code: (i) −*x*+1, −*y*+1, −*z*.
